# Supplementary material for: Tailoring Interlayer Charge Transfer Dynamics in 2D Perovskites with Electroactive Spacer Molecules
Source: J Am Chem Soc. 2023 Sep 22;145(39):21330–43. doi: 10.1021/jacs.3c05974 (PMC10557141; doi:10.1021/jacs.3c05974)
Supplement: Supplementary file 1 — ja3c05974_si_001.pdf [file ja3c05974_si_001.pdf]

Tailoring Interlayer Charge Transfer Dynamics in 2D Perovskites with Electroactive Spacer Molecules

Yorrick Boeije,[1,2†] Wouter T.M. Van Gompel,[3†] Youcheng Zhang,[2,4†] Pratyush Ghosh,[2] Szymon J. Zelewski,[1,2,5] Arthur Maufort,[3] Bart Roose,[1] Zher Ying Ooi,[1] Rituparno Chowdhury,[2] Ilan Devroey,[3] Stijn Lenaers,[3] Alasdair Tew,[2] Linjie Dai,[1,2] Krishanu Dey,[2] Hayden Salway,[1], Richard H. Friend,[2] Henning Sirringhaus,[2] Laurence Lutsen,[3] Dirk Vanderzande,[3] Akshay Rao,[2*] Samuel D. Stranks[1,2*]

[1]Department of Chemical Engineering and Biotechnology, University of Cambridge, Philippa Fawcett Drive, Cambridge, CB3 0AS, UK.

[2]Department of Physics, Cavendish Laboratory, University of Cambridge, JJ Thomson Ave, Cambridge, CB3 0HE, UK.

[3]Hasselt University, Institute for Materials Research (IMO-IMOMEC), Hybrid Materials Design (HyMaD), Martelarenlaan 42, B-3500 Hasselt, Belgium.

[4]Cambridge Graphene Centre, Department of Engineering, University of Cambridge, JJ Thomson Ave, Cambridge, CB3 0FA, UK.

[5]Department of Semiconductor Materials Engineering, Faculty of Fundamental Problems of Technology, Wrocław University of Science and Technology, Wybrzeże Wyspiańskiego 27, 50-370 Wrocław, Poland.

Email: ar525@cam.ac.uk (A.R.), sds65@cam.ac.uk (S. D. S.)

Keywords: Charge transfer, Functionalized 2D perovskites, Organic-inorganic hybrid, Transient absorption spectroscopy, Charge transport, Excitons

Synthesis of organic ammonium salts

PEAI (phenethylammonium iodide) salt was synthesized as follows: phenethyl amine (1.04 mL, 8.25 mmol) was dissolved in ethanol (30 mL), and the flask was subsequently wrapped in aluminium foil. HI (57 wt% in water, unstabilized) was extracted three times with a chloroform/tri-*n*-butyl phosphate mixture (10:1, V/V).[1] Afterwards, the purified HI (1.14 mL, 8.67 mmol) was added to the reaction flask. The resulting mixture was left to stir at room temperature for 2 hours. After 2 h, a portion of ethanol was evaporated under reduced pressure. The residue was then precipitated in a large amount of cooled diethyl ether. The precipitate was collected by filtration and washed several times with diethyl ether. The product was obtained as a white solid (1.65 g, 6,62 mmol; 80% yield). ^{1}H NMR (400 MHz, DMSO-d_6) δ 7.74 (s (br), 3H), 7.37-7.32 (m, 2H), 7.28-7.25 (m, 3H), 3.07-3.02 (m, 2H), 2.88-2.82 (m, 2H).

Cz-C_3I (3-(9*H*-carbazol-9-yl)propylammonium iodide) salt was synthesized as follows:

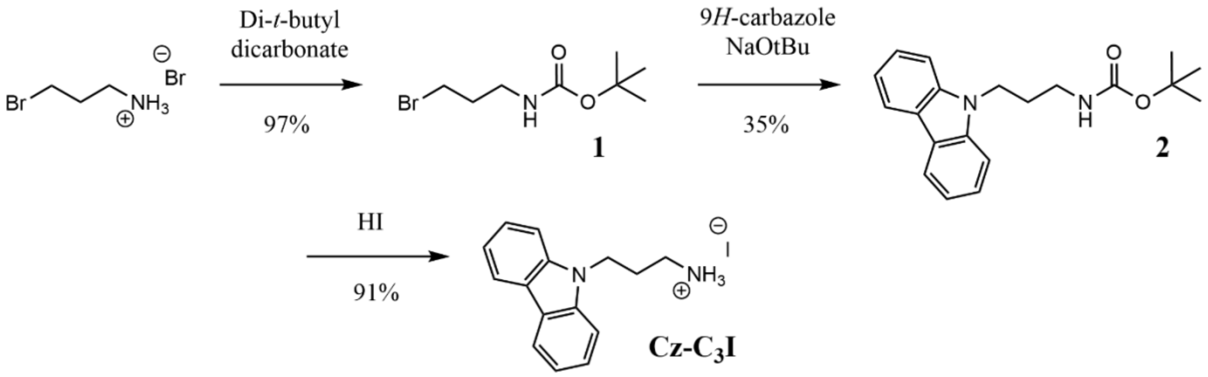

3-bromopropylamine, Boc protected (1)

3-bromopropylammonium bromide (5.00 g, 22.8 mmol) was dissolved in 100 mL dichloromethane and 3.50 mL triethylamine. Di-*t*-butyl dicarbonate (4.23 g, 19.4 mmol) was then added in one portion, after which carbon dioxide gas bubbles formed. The flask was sealed with a septum, which was perforated with two needles to allow carbon dioxide to escape. After 24 h at ambient temperature, the reaction mixture was extracted with water. The organic fraction was then dried, filtered, and concentrated to yield the target compound as a colourless oil (4.49 g, 97% yield). ^{1}H NMR (400 MHz, Chloroform-*d*) δ 4.66 (s, 1H), 3.44 (t, J = 6.5 Hz, 2H), 3.27 (m, J = 6.5 Hz, 2H), 2.05 (p, J = 6.5 Hz, 2H), 1.44 (s, 9H). ^{13}C NMR (101 MHz, Chloroform-*d*) δ 156.07, 79.50, 39.05, 32.76, 30.93, 28.47. GC-MS: m/z = 237 & 239 (target) and 181 & 183 (Boc deprotected target).

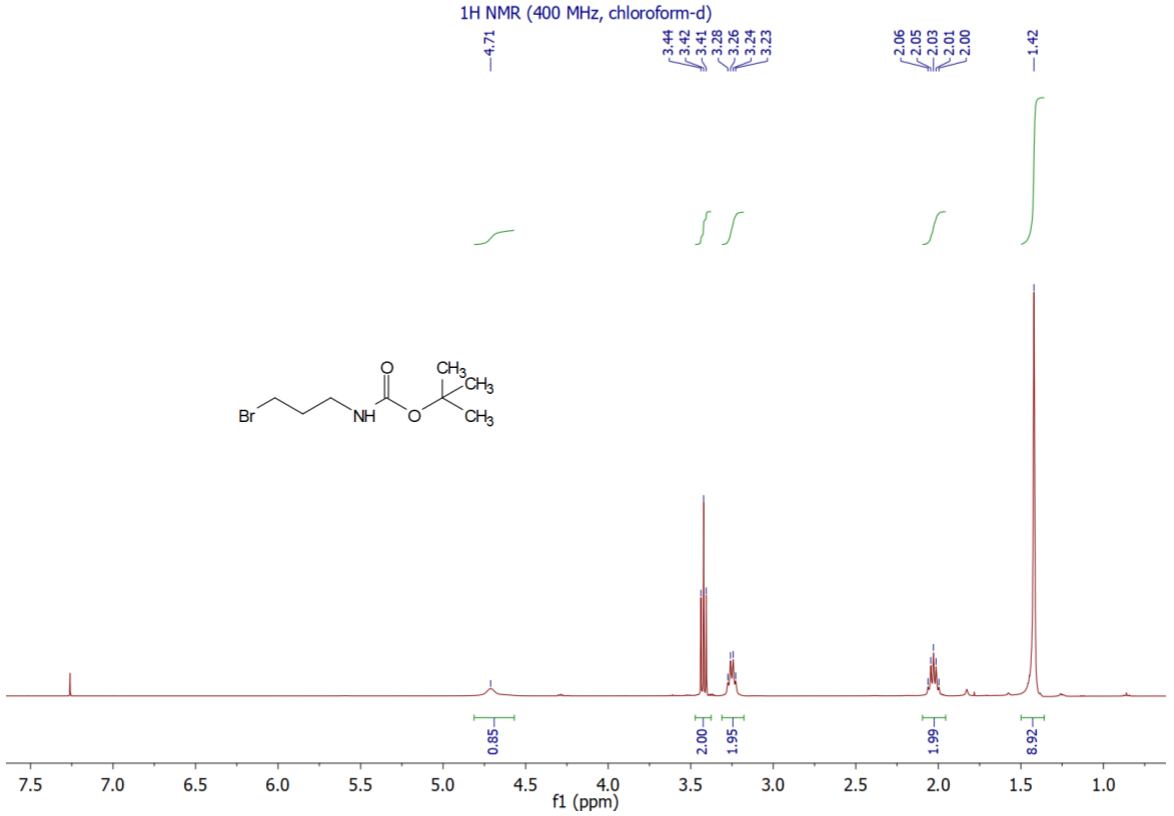

^{1}H NMR spectrum of 3-bromopropylamine, Boc protected (1) (residual internal $CHCl_3$ at 7.26 ppm).

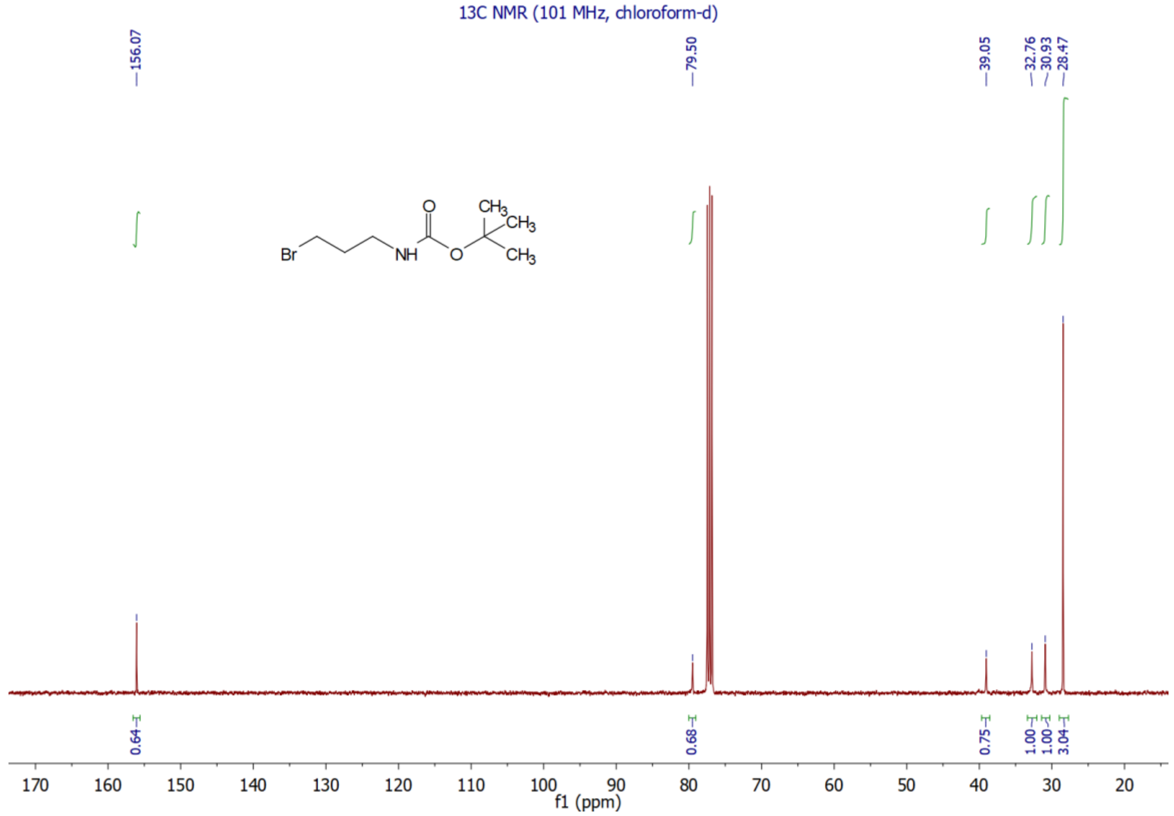

^{13}C NMR spectrum of 3-bromopropylamine, Boc protected (1) (solvent signal: $CDCl_3$ at 77 ppm).

3-(9*H*-carbazol-9-yl)propylamine, Boc protected (2)

9*H*-carbazole (1.99 g, 12.0 mmol) and sodium *t*-butoxide (1.17 g, 12.0 mmol) were dissolved in 25 mL dry THF under Ar atmosphere. **1** (7.15 g, 29.9 mmol) was added, and the resulting mixture was left to react at 55°C under Ar for 6 h. The mixture was then quenched with 1 M NH_4Cl and extracted with chloroform. The chloroform fractions were washed with water, dried, filtered, and concentrated. The resulting crude compound was purified through column chromatography (gradient elution from chloroform to chloroform/ethyl acetate 9:1) and vacuum distillation (Kugelrohr) to yield the target compound as a white powder (1.37 g, 35% yield). ^{1}H NMR (400 MHz, Chloroform-*d*) δ 8.10 (dt, J = 7.7, 1.0 Hz, 2H), 7.47 (ddd, J = 8.2, 7.0, 1.2 Hz, 2H), 7.40 (dt, J = 8.2, 0.9 Hz, 2H), 7.23 (ddd, J = 8.0, 7.0, 1.1 Hz, 2H), 4.51 (s, 1H), 4.37 (t, J = 7.0 Hz, 2H), 3.15 (m, J = 7.3 Hz, 2H), 2.09 (p, J = 7.0 Hz, 2H), 1.43 (s, 9H). ^{13}C NMR (101 MHz, Chloroform-*d*) δ 156.09, 140.31, 125.88, 123.01, 120.56, 119.09, 108.58, 79.53, 40.54, 38.62, 29.34, 28.48. GC-MS: m/z = 224 (Boc deprotected target).

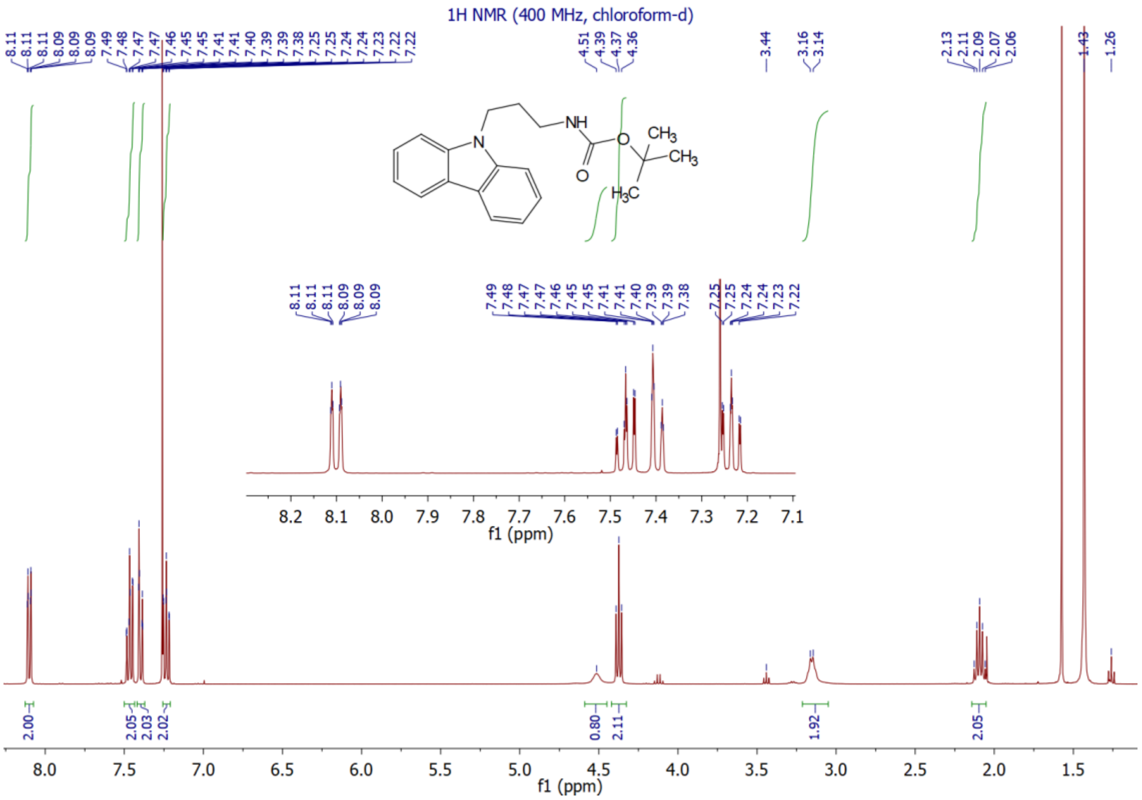

^{1}H NMR spectrum of 3-(9*H*-carbazol-9-yl)propylamine, Boc protected (2) (residual internal $CHCl_3$ at 7.26 ppm; water at 1.56 ppm; ethyl acetate at 4.12 ppm, 2.03 ppm, and 1.25 ppm).

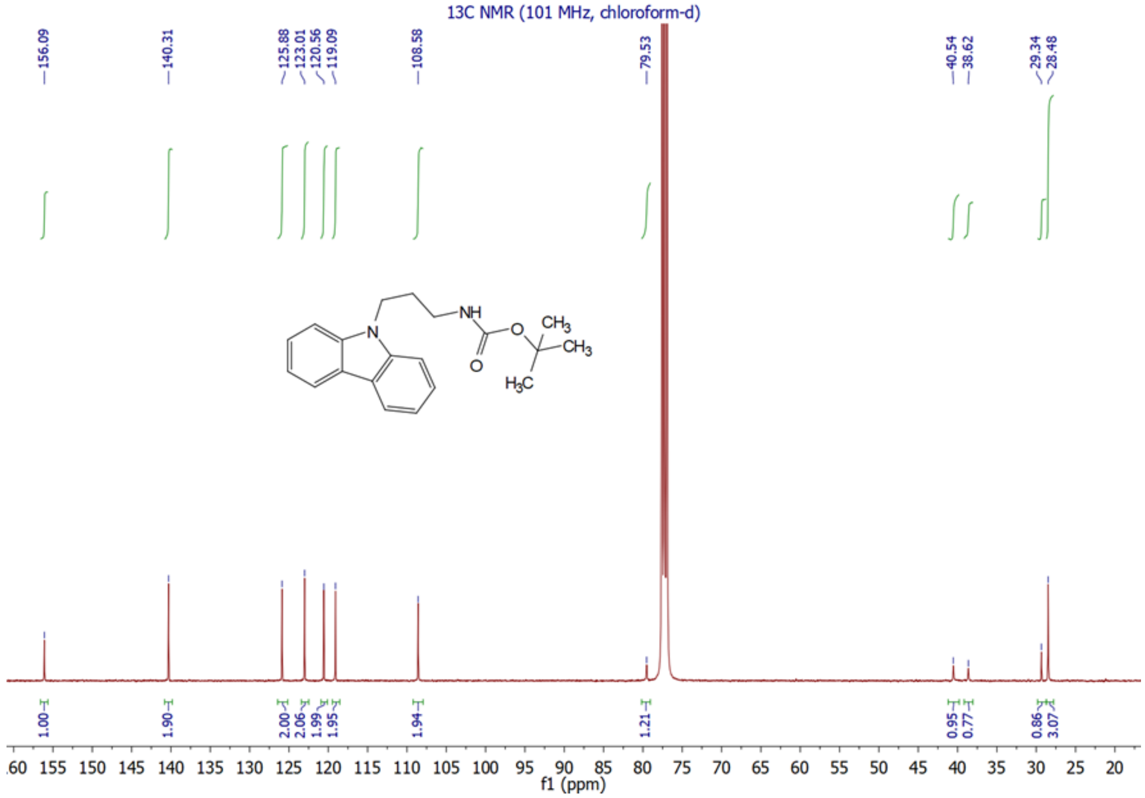

^{13}C NMR spectrum of 3-(9*H*-carbazol-9-yl)propylamine, Boc protected (2) (solvent signal: $CDCl_3$ at 77 ppm).

3-(9*H*-carbazol-9-yl)propylammonium iodide (Cz-C_3I)

HI (57% in water, unstabilized) was extracted three times with a 9:1 mixture of chloroform and tri-*n*-butyl phosphate to remove impurities. 895 µL (6.78 mmol) of this freshly extracted HI was then added with a micropipette to a solution of **2** (1.00 g, 3.08 mmol) in 20 mL dioxane at 40°C under Ar atmosphere. After stirring for 22 h in the dark, the solvent was partially evaporated, and the remaining solution was added dropwise to 400 mL diethyl ether. The target compound precipitates and was separated after filtering under vacuum and washing with a copious amount of diethyl ether. 0.98 g (91% yield) was obtained as a yellow powder. It was dried under high vacuum before further use. ^{1}H NMR (400 MHz, DMSO-d_6) δ 8.17 (dt, J = 7.7, 1.0 Hz, 2H), 7.67 (dt, J = 8.2, 0.9 Hz, 2H), 7.61 (s, 3H), 7.48 (ddd, J = 8.3, 7.1, 1.2 Hz, 2H), 7.22 (ddd, J = 7.9, 7.1, 0.9 Hz, 2H), 4.50 (t, J = 6.9 Hz, 2H), 2.87 – 2.77 (m, 2H), 2.05 (dt, J = 14.6, 7.1 Hz, 2H). ^{13}C NMR (101 MHz, Chloroform-*d*) δ 140.39, 126.35, 122.66, 120.99, 119.52, 37.54, 27.39.

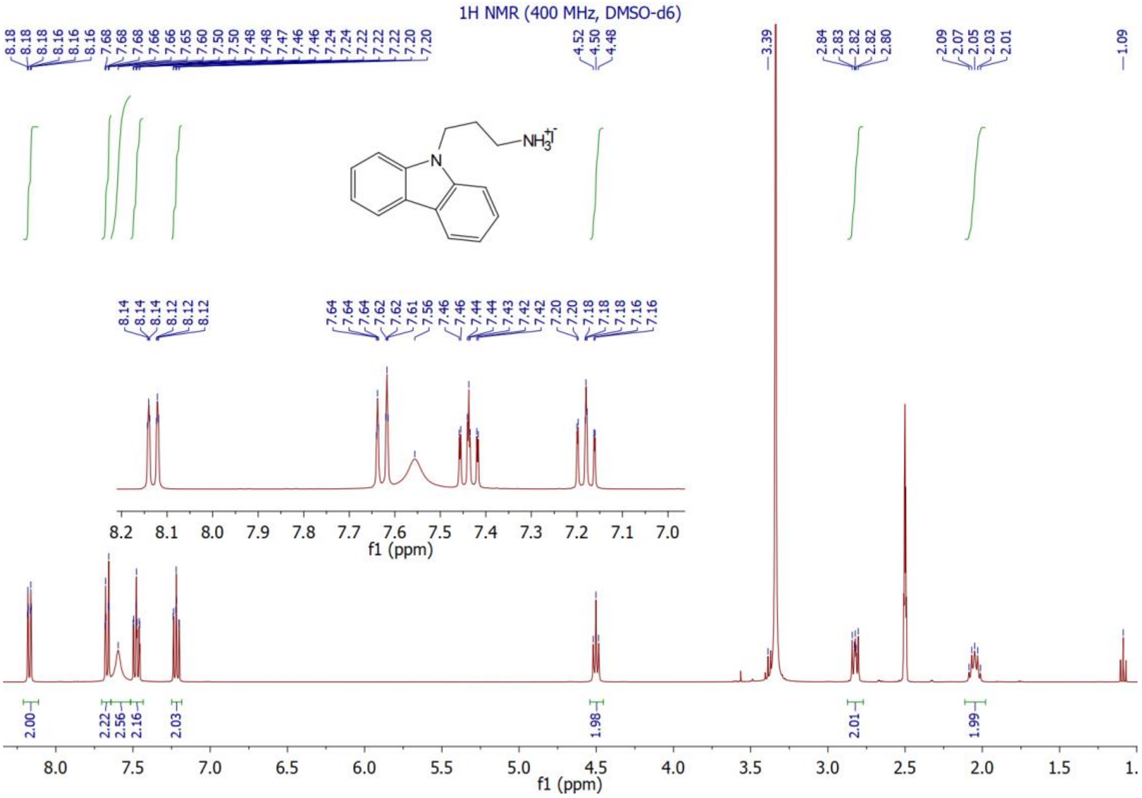

^{1}H NMR spectrum of 3-(9*H*-carbazol-9-yl)propylammonium iodide (Cz-C_3I) (residual internal $(CD_3)(CD_2H)SO$ at 2.50 ppm; water at 3.34 ppm; diethylether at 3.39 ppm and 1.09 ppm).

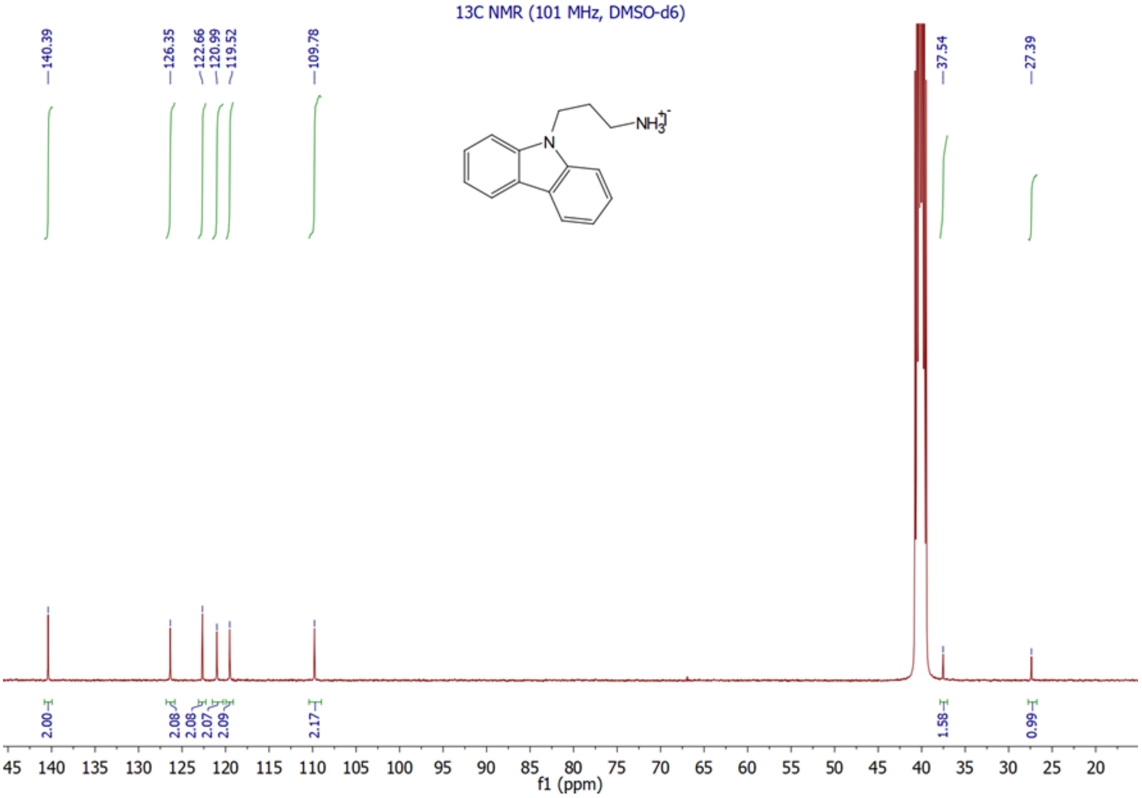

^{13}C NMR spectrum of 3-(9*H*-carbazol-9-yl)propylammonium iodide (Cz-C_3I) (solvent signal: $(CD_3)_2SO$ at 40 ppm).

The Cz-C_4I and Cz-C_5I salts were synthesized according to previous work by some of the authors.[2,3]

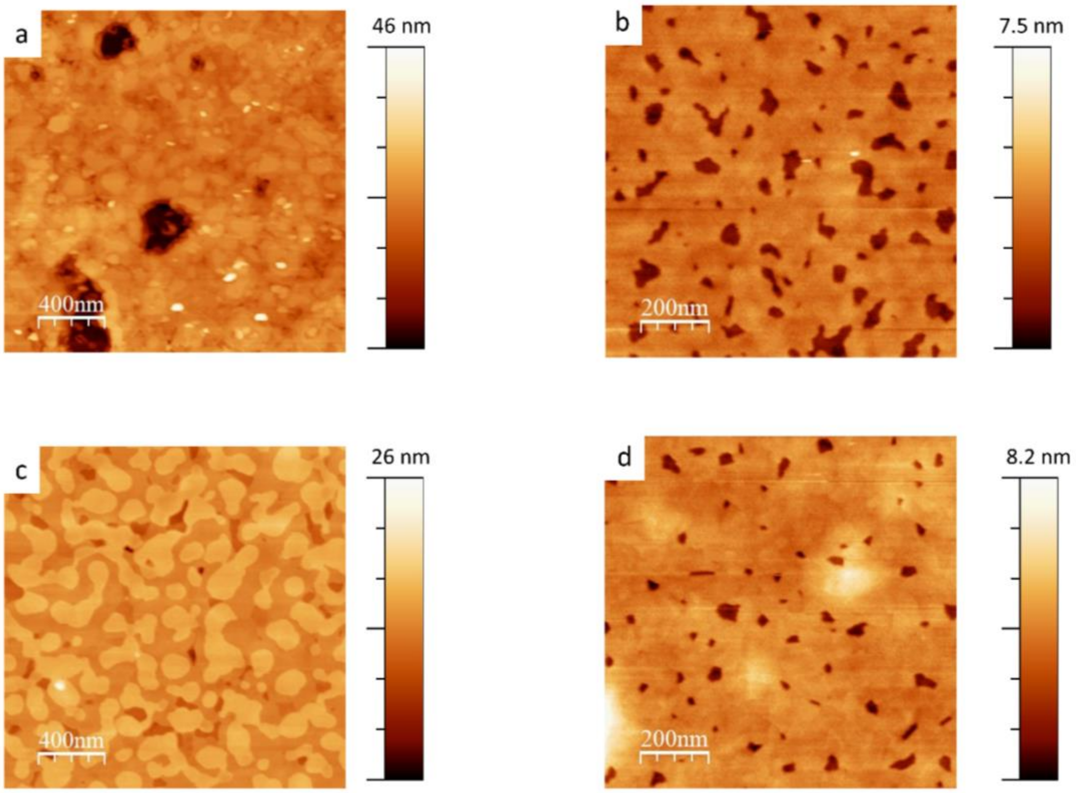

Figure S1 Atomic force microscopy images of $(PEA)_2PbI_4$ (a), $(Cz\text{-}C_3)_2PbI_4$ (b), $(Cz\text{-}C_4)_2PbI_4$ (c) and $(Cz\text{-}C_5)_2PbI_4$ (d). Thicknesses (w) are 17 nm, 38 nm, 34 nm and 46 nm, respectively.

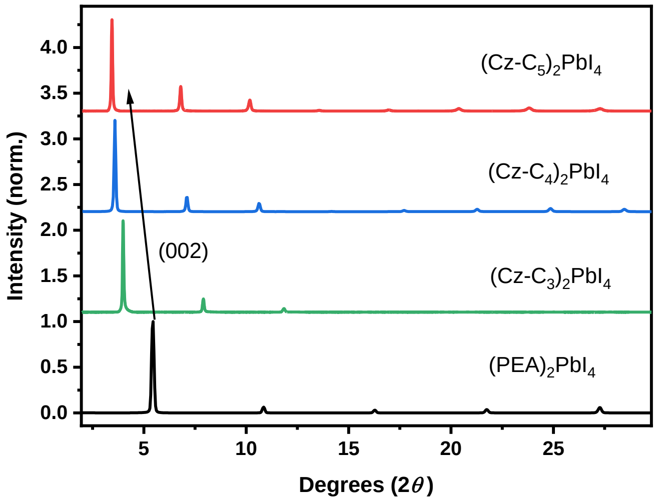

Figure S2 X-ray diffraction patterns of $(PEA)_2PbI_4$, $(Cz\text{-}C_3)_2PbI_4$, $(Cz\text{-}C_4)_2PbI_4$ and $(Cz\text{-}C_5)_2PbI_4$ thin films. The arrow indicates that the position of the (002) reflection shifts to lower diffraction angle (2θ) with increasing alkyl spacer length, indicating an increase in interplanar spacing (d-spacing).

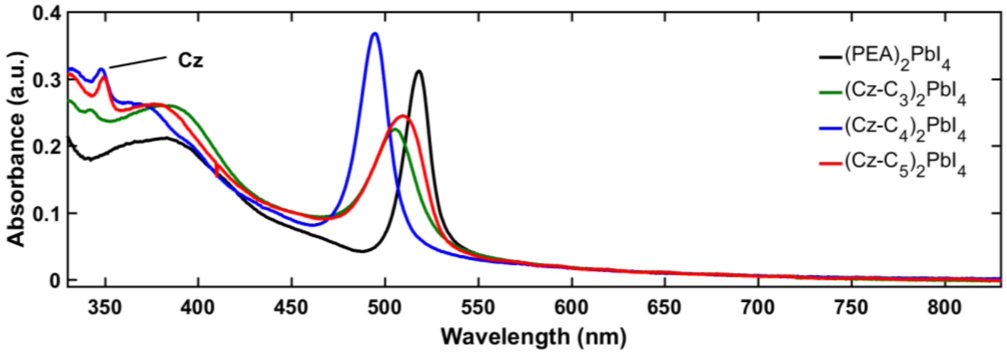

Figure S3 UV/VIS absorption spectra of $(PEA)_2PbI_4$, $(Cz\text{-}C_3)_2PbI_4$, $(Cz\text{-}C_4)_2PbI_4$ and $(Cz\text{-}C_5)_2PbI_4$ thin films. The first excited state of Cz-C_n is indicated with a bar. Note the small spectral shifts.

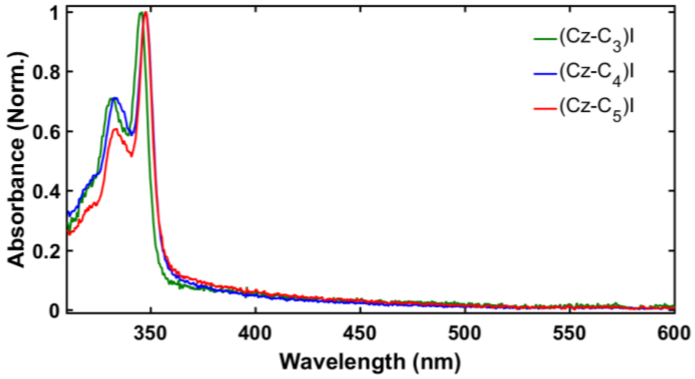

Figure S4 Normalized UV/VIS Absorption spectra of the carbazole alkyl ammonium salts.

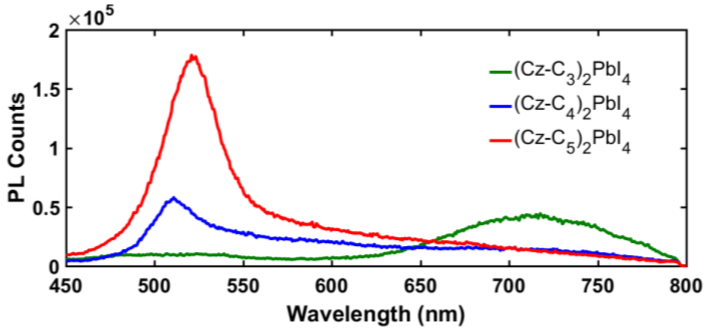

Figure S5 Photoluminescence spectra of $(Cz\text{-}C_3)_2PbI_4$, $(Cz\text{-}C_4)_2PbI_4$ and $(Cz\text{-}C_5)_2PbI_4$ thin films. λ_{exc} = 400 nm (0.8 mJ/cm^2).

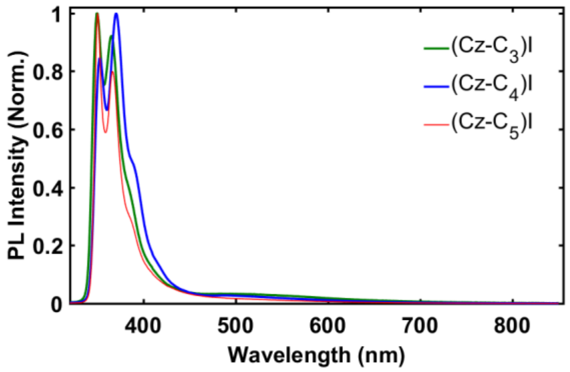

Figure S6 Normalized photoluminescence spectra of the carbazole alkyl ammonium salts, excited at 300 nm.

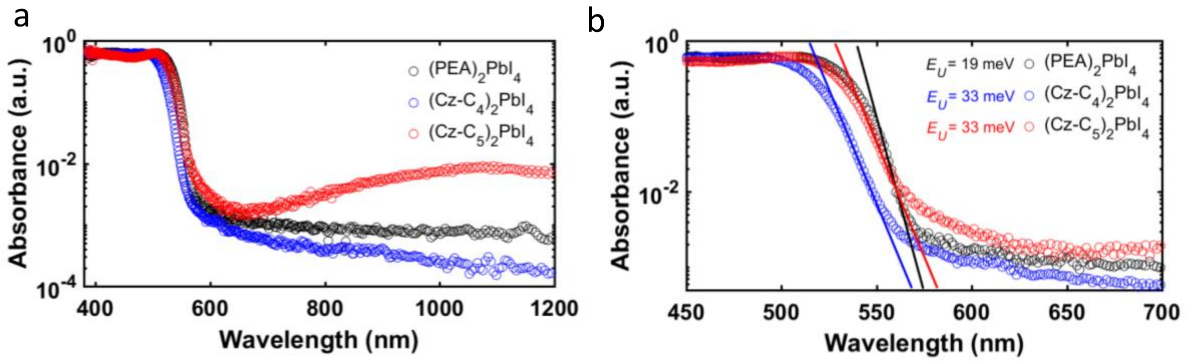

Figure S7 (a) Normalized photothermal deflection spectra of PEA_2PbI_4, $(Cz\text{-}C_4)_2PbI_4$ and $(Cz\text{-}C_5)_2PbI_4$ thin films. (b) zoom-in on the sub-gap absorption tail. Urbach energies are extracted from the slope.

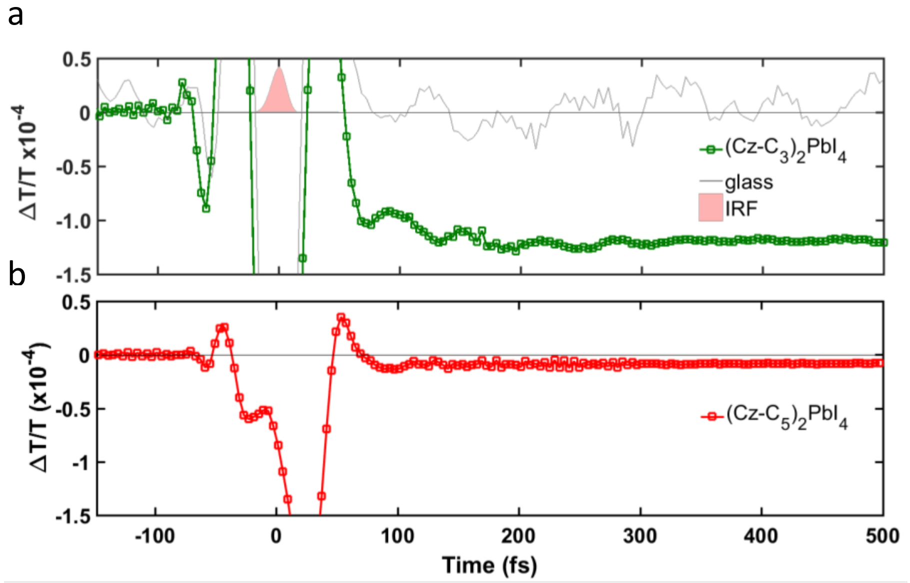

Figure S8 Femtosecond transient absorption spectroscopy of $(Cz\text{-}C_3)_2PbI_4$ (a) and $(Cz\text{-}C_5)_2PbI_4$ (b) thin films, showing the rise kinetics of the same spectral region (790-820 nm) that was investigated for $(Cz\text{-}C_3)_2PbI_4$ in Figure 2 of the main text. The same pump pulse, also shown in this figure, was used. The instrument response function (IRF) is determined by the temporal width of this pulse, which is shown in (a).

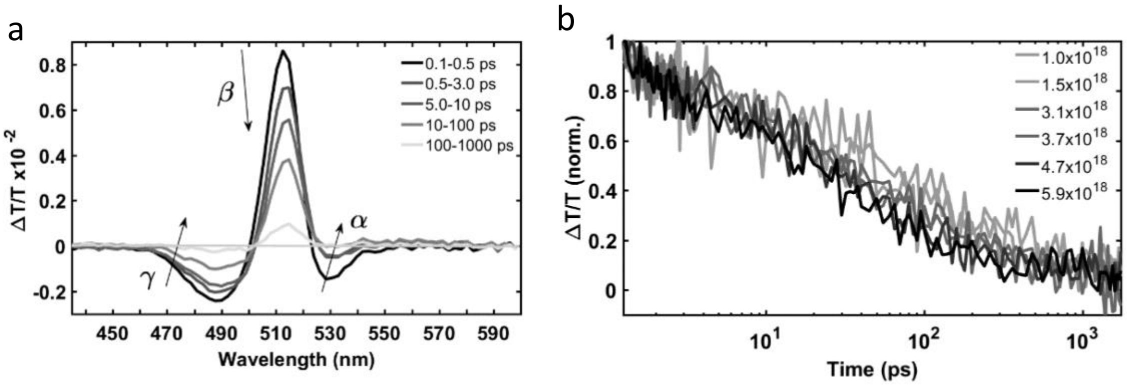

Figure S9 (a) Transient absorption spectra of a $(PEA)_2PbI_4$ thin film excited at 400 nm (5.6 μJ/cm^2) integrated over different time regimes. The labels α, β and γ are discussed in the main text. (b) Kinetics of the exciton bleach (510-520 nm, β) of $(PEA)_2PbI_4$ thin film excited at 400 nm with different carrier densities (cm^{-3}), normalized at 1 ps.

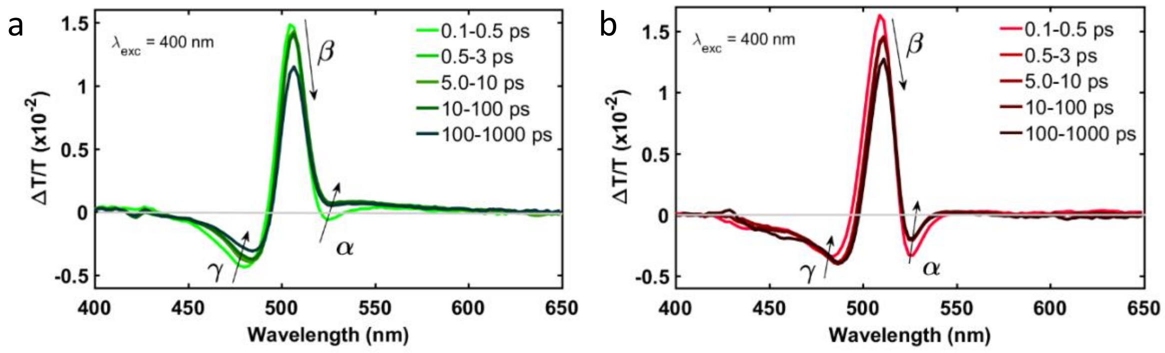

Figure S10 Transient absorption spectra of $(Cz\text{-}C_3)_2PbI_4$ (a) and $(Cz\text{-}C_5)_2PbI_4$ (b) thin films excited at 400 nm integrated over different time regimes. The fluences (carrier densities) were 9.3 μJ/cm^2 ($1.6\cdot10^{18}$ cm^{-3}) and 4.2 μJ/cm^2 ($6.5\cdot10^{17}$ cm^{-3}), respectively. The labels α, β and γ are discussed in the main text.

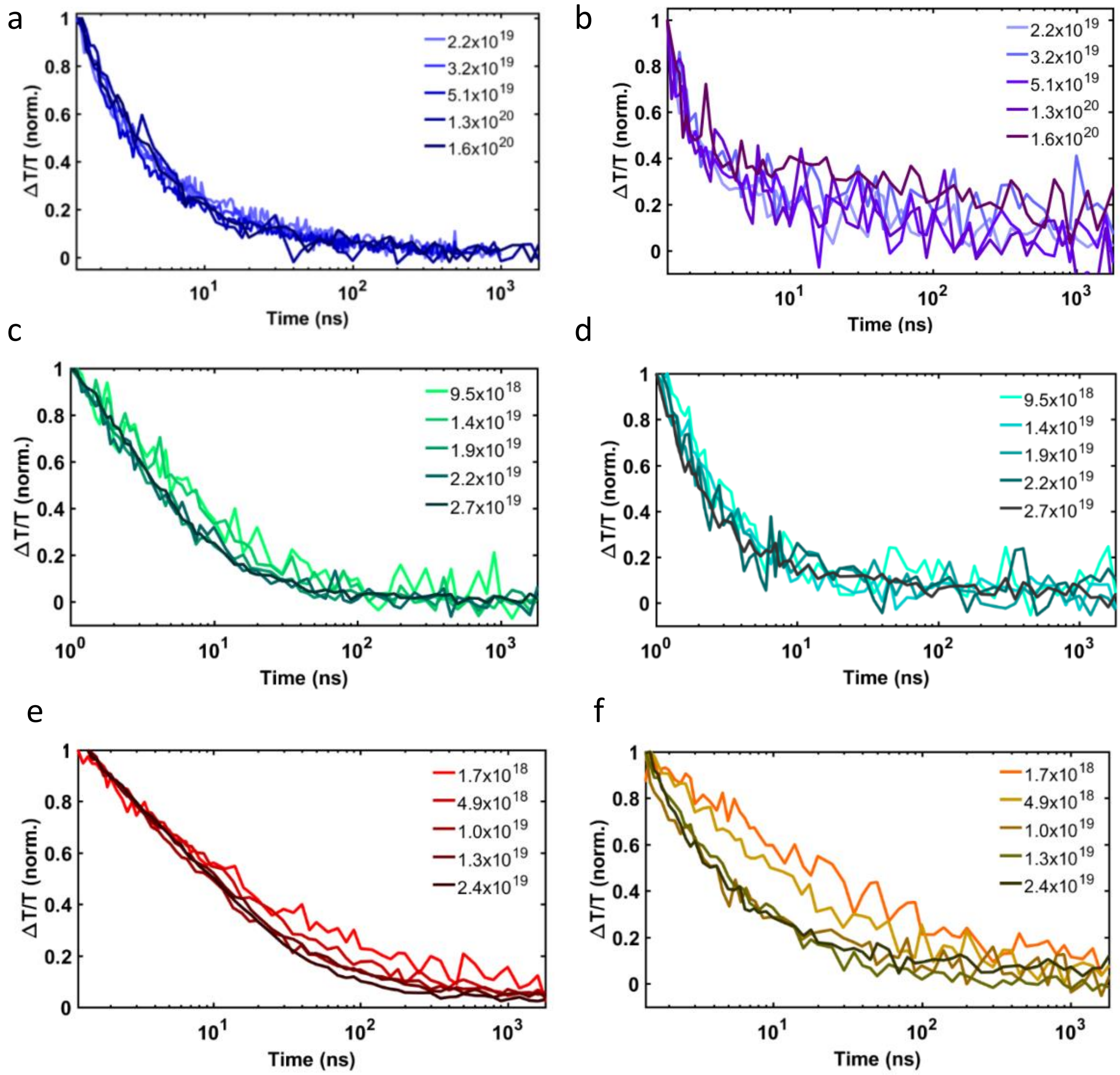

Figure S11 Normalized nanosecond kinetics of the exciton bleach (β) and photoinduced absorption (Cz^+) for $(Cz\text{-}C_4)_2PbI_4$ (a,b), $(Cz\text{-}C_3)_2PbI_4$ (c,d) and $(Cz\text{-}C_5)_2PbI_4$ (e,f) thin films excited at 400 nm with different carrier densities (cm^{-3}).

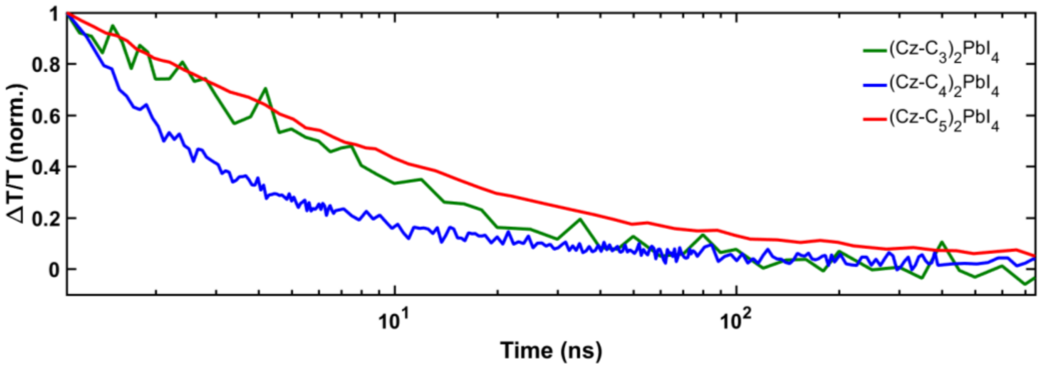

Figure S12 Normalized nanosecond kinetics of the exciton bleach (β) for $(Cz\text{-}C_4)_2PbI_4$, $(Cz\text{-}C_3)_2PbI_4$ and $(Cz\text{-}C_5)_2PbI_4$ thin films excited at 400 nm (1×10^{19} cm^{-3}).

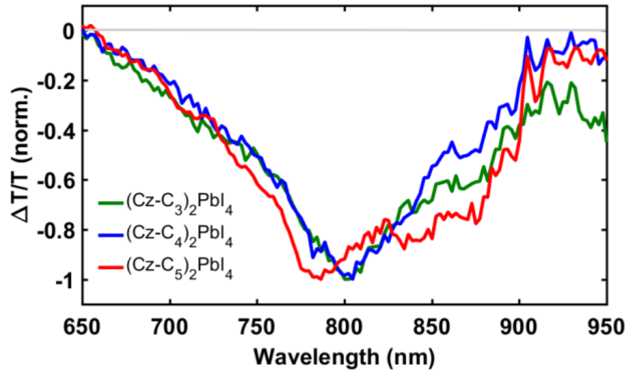

Figure S13 Normalized transient absorption spectra for $(Cz\text{-}C_3)_2PbI_4$, $(Cz\text{-}C_4)_2PbI_4$ and $(Cz\text{-}C_5)_2PbI_4$ thin films excited at their 1s transition (495 nm for $(Cz\text{-}C_4)_2PbI_4$ and 505 nm for $(Cz\text{-}C_3)_2PbI_4$ and $(Cz\text{-}C_5)_2PbI_4$ with carrier densities of 1×10^{19} cm^{-3} and integrated from 1-3 ps.

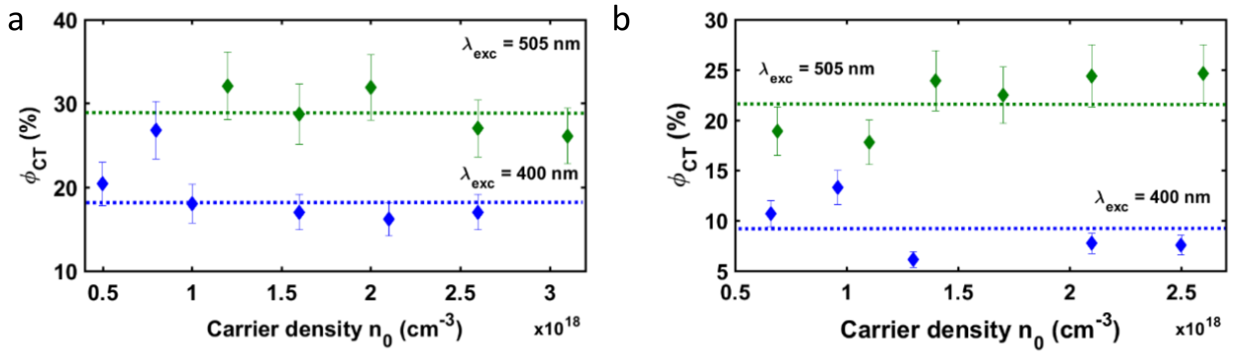

Figure S14 Charge transfer quantum yields φ_{CT} for $(Cz\text{-}C_3)_2PbI_4$ and $(Cz\text{-}C_5)_2PbI_4$ thin films excited at 400 nm and their 1s transition (505 nm) with different carrier densities. The dotted lines indicate the average φ_{CT} values which are used in Fig. 3b.

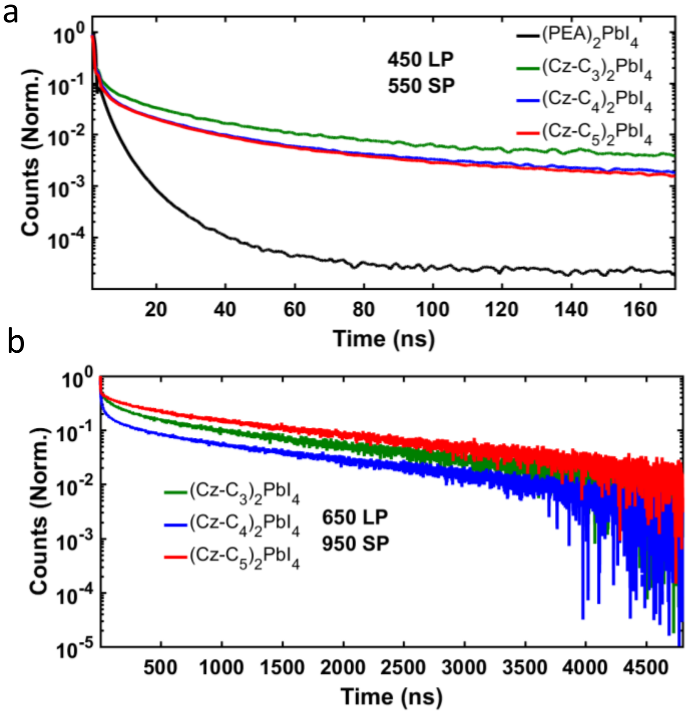

Figure S15 Normalized time-correlated single photon counting decay curves of $(PEA)_2PbI_4$ and $(Cz\text{-}C_i)_2PbI_4$ films photoexcited at 407 nm (0.10 μJ/cm²) using two different sets of filters to selectively detect either the blue (a) or red (b) emission (see Figure S5).

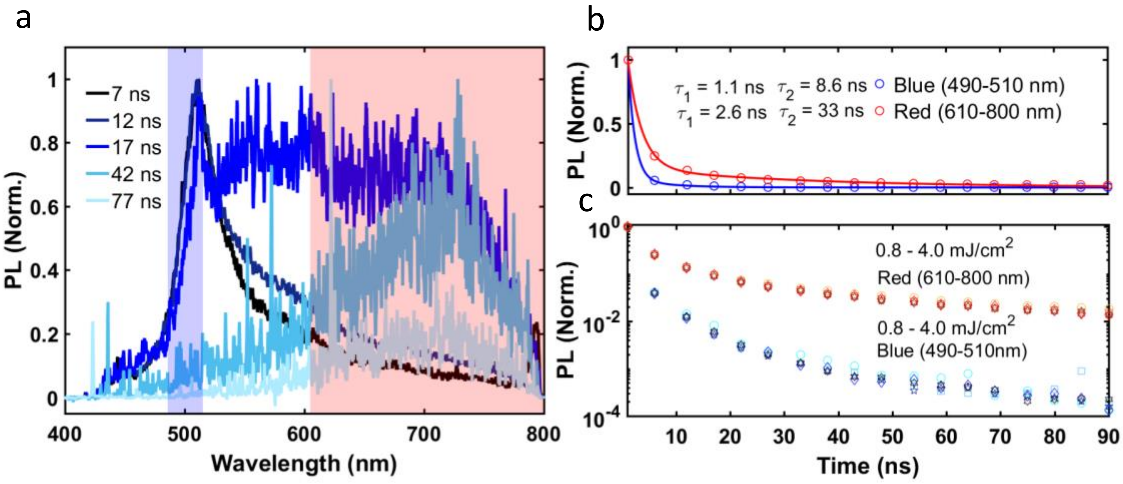

Figure S16 (a) Normalized time-resolved photoluminescence spectra of a $(Cz\text{-}C_4)_2PbI_4$ thin film photoexcited at 400 nm (0.8 mJ/cm²). (b) Nanosecond photoluminescence kinetics of blue and red spectral regions, which are indicated with the shaded boxes in (a), and their corresponding bi-exponential fits. (c) Fluence dependent photoluminescence kinetics of blue and red spectral regions, 0.8 mJ/cm², 1.6 mJ/cm², 2.4 mJ/cm², 3.2 mJ/cm², 4.0 mJ/cm².

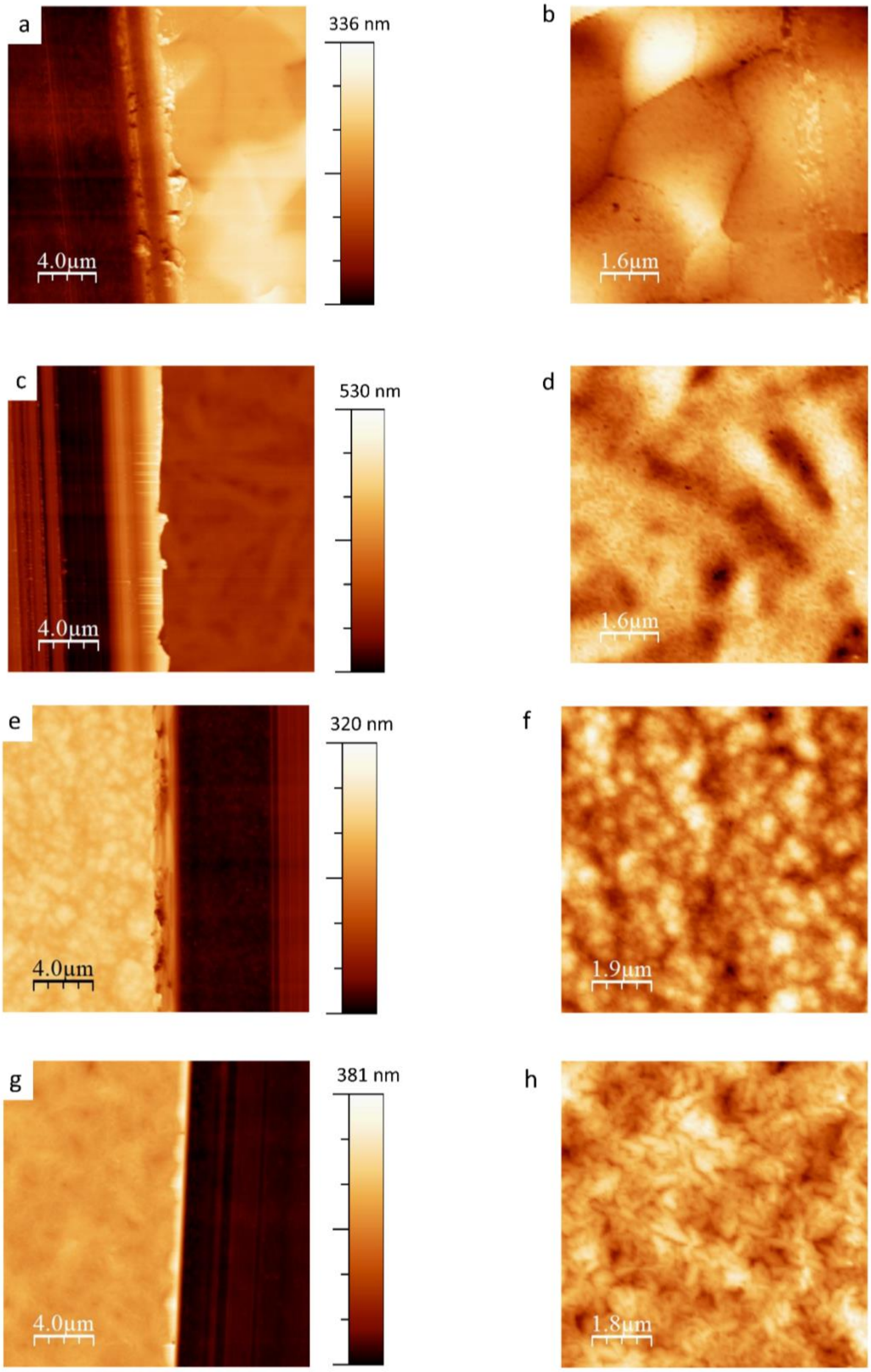

Figure S17 Atomic Force Microscopy images of $(PEA)_2PbI_4$ (a-b), $(Cz\text{-}C_3)_2PbI_4$ (c-d) $(Cz\text{-}C_4)_2PbI_4$ (e-f) and $(Cz\text{-}C_5)_2PbI_4$ (g-h) films spin-coated on ITO substrates. Averaged thicknesses of multiple films are provided in Table S2.

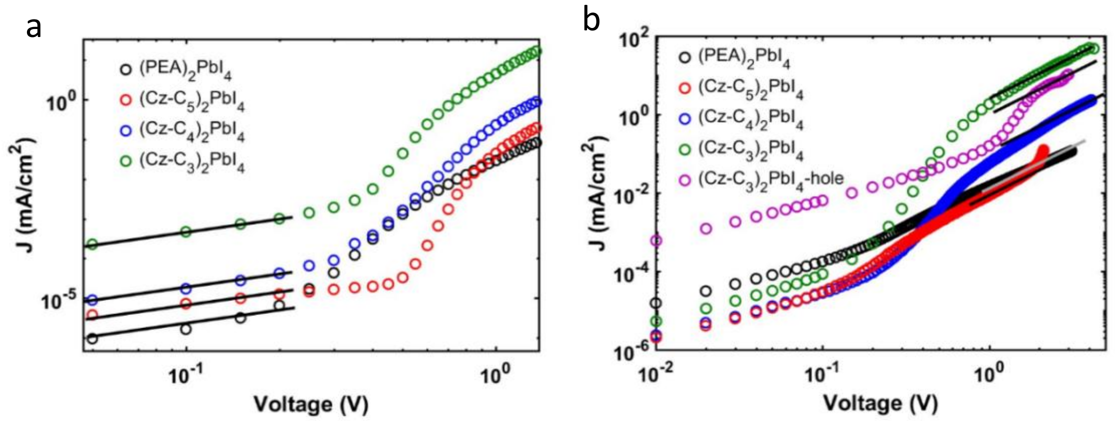

Figure S18 Representative J-V curves across multiple voltage regimes measured on several 2D perovskite vertical transport devices (shown in Figure 5 of the main text). (a) ambipolar devices used for out-of-plane conductivity (σ_{OOP}) determination. The black lines indicate the linear (ohmic) regime. (b) electron-selective devices used for out-of-plane mobility (μ_{OOP}) determination. The black lines (grey for $(PEA)_2PbI_4$) indicate the quadratic (SCLC) regime. The J-V curve of the hole-selective device for $(Cz\text{-}C_3)_2PbI_4$ is shown as well

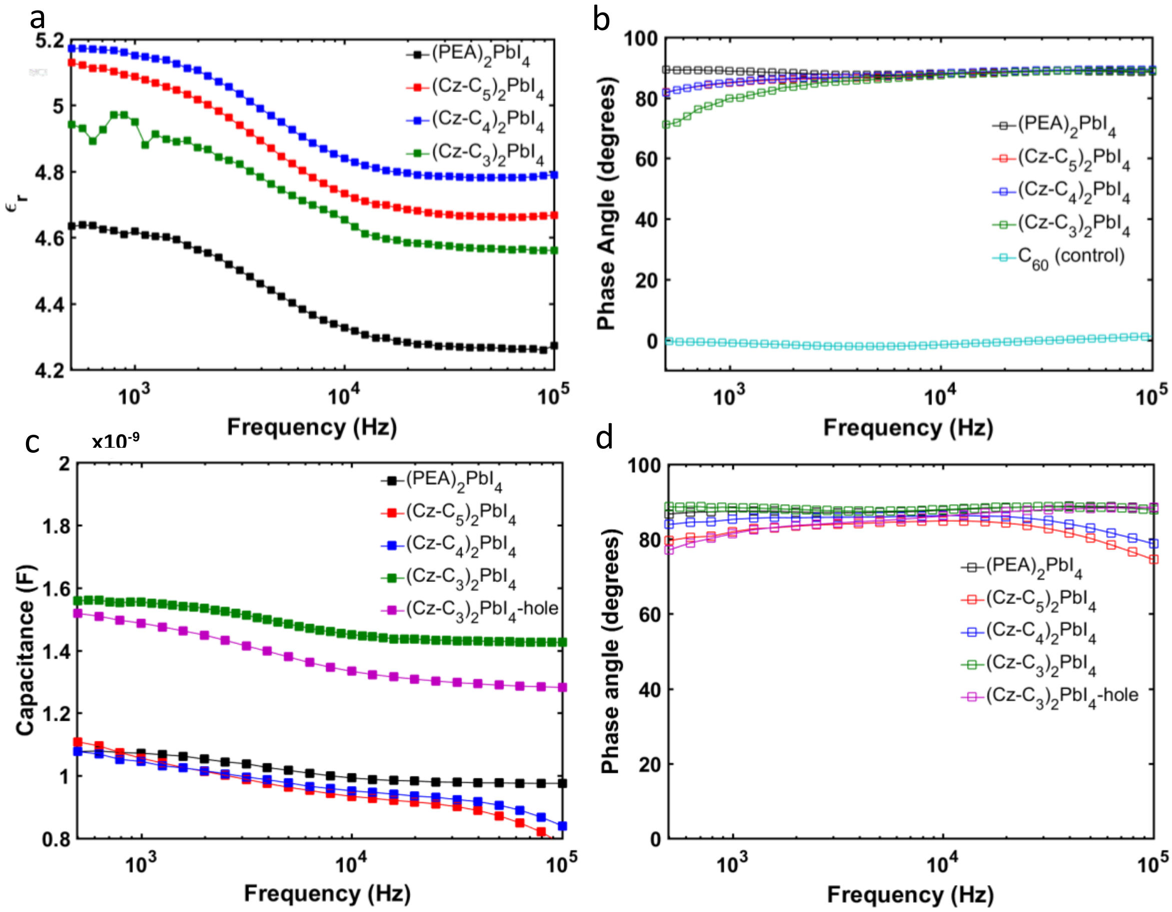

Figure S19 (a) relative dielectric constant (ε_r) of the 2D perovskite ambipolar devices (Figure 5 main text) by capacitance-frequency (C-F) measurement using the thickness determined from AFM, see Table S2. (b) Phase angle curves of the C-F measurement for the same devices and a Au/C_{60}(20 nm)/ITO control device. (c) C-F measurement for electron-only transport devices. Capacitance at 500 Hz is used for out-of-plane mobility determination. (d) Phase angle curves of the C-F measurements for the same devices.

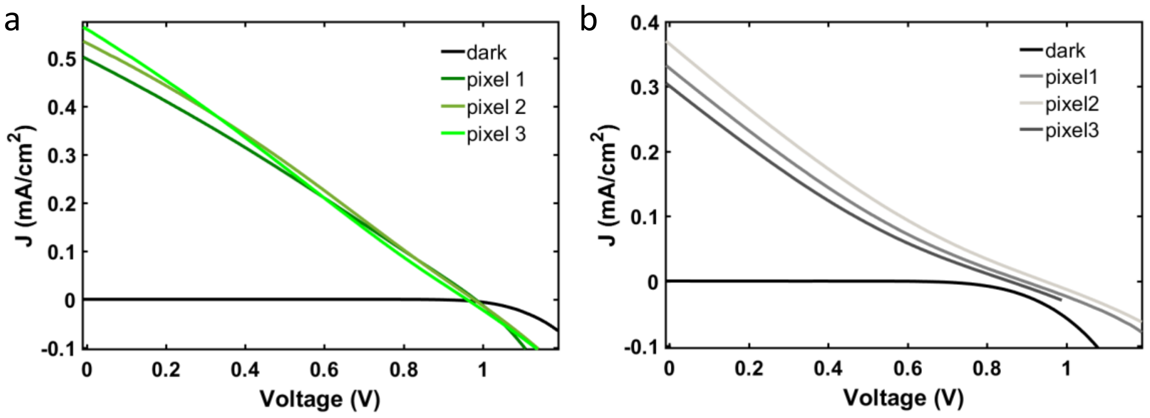

Figure S20 J-V curves for a photovoltaic device of $(Cz\text{-}C_3)_2PbI_4$ (a) and $(PEA)_2PbI_4$ (b). Corresponding parameters are provided in Table S1.

Table S1. Extracted photovoltaic parameters of J-V curves shown in Figure S20.

	V_{OC} (V)	J_{SC} (mA/cm^2)	FF (%)	PCE (%)
Pixel 1 $(Cz\text{-}C_3)_2PbI_4$	0.980	0.502	26.8	0.132
Pixel 2 $(Cz\text{-}C_3)_2PbI_4$	0.978	0.535	27.3	0.143
Pixel 3 $(Cz\text{-}C_3)_2PbI_4$	0.959	0.564	25.3	0.137
Pixel 1 $(PEA)_2PbI_4$	0.888	0.333	19.6	0.058
Pixel 2 $(PEA)_2PbI_4$	0.943	0.370	19.8	0.069
Pixel 3 $(PEA)_2PbI_4$	0.858	0.305	19.5	0.051

S1 Determination of charge transfer quantum yield and experimental error

The charge transfer quantum yield, denoted as φ_{CT}, is defined as the ratio of $Cz^{+}\cdot$ molecules generated per unit volume to the number of absorbed photons per unit volume, n_0:

$$\varphi_{CT}\ (\%) = \frac{Cz^{+}\cdot}{n_0} \times 100 \qquad \text{(S1)}$$

$Cz^{+}\cdot$ (cm^{-3}) is related to the TA signal at a certain probe wavelength and time delay, $\frac{\Delta T}{T}$, the absorption coefficient at the same probe wavelength, σ (cm^2), and the film thickness, w (cm), through

$$\frac{\Delta T}{T} = -w \times \sigma \times Cz^{+}\cdot \qquad \text{(S2)}$$

We use AFM to measure w (Figure S1, SI) and we take σ from ref. 4. To calculate n_0 and to correct for the different sizes in pump and probe pulses, we follow the procedure of ref. 5, which takes into account the Gaussian shapes of the pulses. The improved n_0 is calculated through equation S3:

$$n_0 = \frac{F_{corr}\left(1-10^{-A}\right)}{wE_{photon}} \qquad \text{(S3)}$$

where F is the pump fluence, A is the absorbance at the pump wavelength, w is the film thickness (this expression is valid since the absorption depth >> film thickness) and E_{photon} is the photon energy of the pump. The estimated amount of $Cz^{+}\cdot$ absorption determined from the probe beam absorption

assumes the same vertically uniform laser profile. However, the pump and probe beam spot sizes are significantly different in our experiments. As mentioned in the main text, we use the procedure in ref. 5 to correct for this. The pump intensity in the region of pump-probe beam overlap, $I_{pump}(r_{probe})$, is calculated using equation S4

$$I_{pump}(r_{probe}) = I_{tot}\left[1 - \exp\left(\frac{-2r_{probe}^2}{r_{pump}^2}\right)\right] \tag{S4}$$

where I_{tot} is the total intensity of the pump beam on the film, r_{probe} is the probe beam spot size and r_{pump} is the pump beam spot size. The latter two are extracted by taking $1/e^2$ radius of a Gaussian fit. Using this corrected pump intensity, F is computed according to equation S5 and substituted in S1 to calculate φ_{CT}.

$$F_{corr} = \frac{I_{pump}(r_{probe})}{(\pi \times r_{probe}^2) \times f} \tag{S5}$$

in which f is the repetition rate of the laser.

The total experimental errors are mainly caused by errors in I_{tot}, r_{probe} and r_{pump}. We use error propagation schemes based on relative errors of 6%, 3% and 5% for I_{tot}, r_{probe} and r_{pump}, respectively, to yield the error of 13% in $I_{pump}(r_{probe})$. The absolute charge transfer quantum yield values are multiplied by this relative error to yield the absolute experimental error.

S2 Determination of out-of-plane conductivity and charge carrier mobility and experimental error

Out-of-plane conductivities (σ_{OOP}) were determined from the J-V curves in the ohmic regime, where there is a linear relationship between current density and voltage. The σ_{OOP} is determined by multiplying the slope, $a = \left(\frac{dJ}{dV}\right)$, with the perovskite film thickness L, as determined by AFM. The error in σ_{OOP} has contributions from both as expressed in Equation S6:

$$\left(\frac{\Delta\sigma}{\sigma}\right)^2 = \left(\frac{\Delta a}{a}\right)^2 + \left(\frac{\Delta L}{L}\right)^2 \tag{S6}$$

Out-of-plane mobilities (μ_{OOP}) were determined from the J-V curves in the space-charge limited current (SCLC) regime, where there is a quadratic relationship between the current density and voltage. This is expressed in the Mott-Gurney equation as:

$$J = \frac{9}{8} * \varepsilon * \mu * \frac{V^2}{L^3} \tag{S7}$$

where J is the current density, ε is the dielectric constant, μ is the mobility, L is the perovskite film thickness.

$J = I/A$ and $C_{perovskite} = \frac{\varepsilon A}{L}$, where A is the area of the device pixel. As we are using the same device pixel for the I-V and capacitance measurements, the area term A may be cancelled by the following transformation:

$$\frac{I}{A} = \frac{9}{8} * \left(\frac{C_{perovskite} * L}{A}\right) * \mu * \frac{V^2}{L^3} \tag{S8}$$

$$I = \frac{9}{8} * C_{perovskite} * \mu * L^{-2} * V^2 \tag{S9}$$

And we finally obtain the equation for the extraction of mobility after some further rearranging:

$$\sqrt{I} = (\frac{9}{8} * C_{perovskite} * \mu * L^{-2})^{1/2} * V \quad \text{(S10)}$$

$$\left(\frac{d\sqrt{I}}{dV}\right)^2 = \frac{9}{8} * C_{perovskite} * \mu * L^{-2} \quad \text{(S11)}$$

$$\mu = \frac{8}{9} * \left(\frac{d\sqrt{I}}{dV}\right)^2 * \frac{L^2}{C_{perovskite}} \quad \text{(S12)}$$

Errors in the extracted mobility have three contributions: 1) SCLC regime slope $k = \frac{d\sqrt{I}}{dV}$, for which the error is the standard deviation of the linear regression (Δk), 2) the error in perovskite film thickness, ΔL, and 3) the error in capacitance, $\Delta C_{perovskite}$, measured with impedance spectroscopy. The standard deviation of mobility ($\Delta\mu$) is then given by the following equation:

$$\left(\frac{\Delta\mu}{\mu}\right)^2 = \left(\frac{2\Delta k}{k}\right)^2 + \left(\frac{2\Delta L}{L}\right)^2 + \left(\frac{-\Delta C_{perovskite}}{C_{perovskite}}\right)^2 \quad \text{(S13)}$$

Finally, there is an error associated with mobilities determined from different *N* devices. The average standard deviation of the mobility $\langle\Delta\mu\rangle$ is determined by the following equation:

$$\langle\Delta\mu\rangle = \sqrt{\frac{1}{N} * \sum_i\left(\mu_i^2 + \Delta\mu_i^2\right) - \left(\frac{1}{N} * \sum_i \mu_i\right)^2} \quad \text{(S14)}$$

All relevant parameters and corresponding errors are provided in Table S2 and S3 for out-of-plane conductivity and mobility determination, respectively.

Table S2. Parameters with corresponding errors used for out-of-plane conductivity determination.

	a (mA/V·cm^2)	L (nm)	σ_{OOP} (S/cm)
$(PEA)_2PbI_4$	2.03(±0.25)x10^{-5}	140±14	2.84(±0.45)x10^{-13}
$(Cz\text{-}C_5)_2PbI_4$	4.48(±0.28)x10^{-5}	208±21	9.32(±1.1)x10^{-13}
$(Cz\text{-}C_4)_2PbI_4$	2.42(±0.31)x10^{-4}	159±16	3.84(±0.63)x10^{-12}
$(Cz\text{-}C_3)_2PbI_4$	6.33(±0.46)x10^{-3}	87±9	5.51(±0.68)x10^{-11}

Table S3. Parameters with corresponding errors used for out-of-plane mobility determination.

	k ($\sqrt{A}/V$)	C (F) x10^{-9}	L (nm)	μ_{OOP} (cm^2/V·s)
$(PEA)_2PbI_4$ electron	7.54(±0.05)x10^{-4}	1.077±0.1	166±15	1.31(±0.48)x10^{-7}
$(Cz\text{-}C_5)_2PbI_4$ electron	1.09(±0.02)x10^{-3}	1.108±0.1	193±8	3.55(±0.45)x10^{-7}
$(Cz\text{-}C_4)_2PbI_4$ electron	3.89(±0.02)x10^{-3}	1.077±0.1	206±10	5.87(±3.62)x10^{-6}
$(Cz\text{-}C_3)_2PbI_4$ electron	1.18(±0.05)x10^{-2}	1.597±0.1	117±14	1.06(±0.30)x10^{-5}
$(Cz\text{-}C_3)_2PbI_4$ hole	6.70(±0.51)x10^{-3}	1.440±0.1	117±14	3.85(±1.28)x10^{-6}

References

(1) Jana, M. K.; Janke, S. M.; Dirkes, D. J.; Dovletgeldi, S.; Liu, C.; Qin, X.; Gundogdu, K.; You, W.; Blum, V.; Mitzi, D. B. Direct-Bandgap 2D Silver-Bismuth Iodide Double Perovskite: The Structure-Directing Influence of an Oligothiophene Spacer Cation. *J Am Chem Soc* **2019**, *141* (19), 7955–7964. DOI:10.1021/JACS.9B02909.

(2) Herckens, R.; Van Gompel, W. T. M.; Song, W.; Gélvez-Rueda, M. C.; Maufort, A.; Ruttens, B.; D'Haen, J.; Grozema, F. C.; Aernouts, T.; Lutsen, L.; Vanderzande, D. Multi-Layered Hybrid Perovskites Templated with Carbazole Derivatives: Optical Properties, Enhanced Moisture Stability and Solar Cell Characteristics. *J. Mater. Chem. A* **2018**, *6* (45), 22899–22908. DOI:10.1039/c8ta08019d.

(3) Van Landeghem, M.; Van Gompel, W. T. M.; Herckens, R.; Lutsen, L.; Vanderzande, D.; Van Doorslaer, S.; Goovaerts, E. Light-Induced Charge Transfer in Two-Dimensional Hybrid Lead Halide Perovskites. *J. Phys. Chem. C* **2021**, *125* (33), 18317–18327. DOI:10.1021/acs.jpcc.1c05005.

(4) Tsujii, Y.; Takami, K.; Tsuchida, A.; Ito, S.; Onogi, Y.; Yamamoto, M. Steric Effect on Dimer Radical Cation Formation of Poly(3,6-Di-Tert-Butyl-9-Vinylcarbazole) and Its Dimeric Model Compounds Studied by Laser Photolysis. *Polym. J.* **1990**, *22* (4), 319–325. DOI:10.1295/polymj.22.319.

(5) Hart, L. J. F.; Grüne, J.; Liu, W.; Lau, T.; Luke, J.; Chin, Y.-C.; Jiang, X.; Zhang, H.; Sowood, D. J. C.; Unson, D. M. L.; Kim, J.-S.; Lu, X.; Zou, Y.; Gao, F.; Sperlich, A.; Dyakonov, V.; Yuan, J.; Gillett, A. J. Triplet-Triplet Annihilation Reduces Non-Radiative Voltage Losses in Organic Solar Cells. *ArXiv* **2023**. DOI:10.48550/arxiv.2301.02112.
